# Mechanistic interplay between ceramide and insulin resistance

**DOI:** 10.1038/srep41231

**Published:** 2017-01-23

**Authors:** Federico Reali, Melissa J. Morine, Ozan Kahramanoğulları, Suryaprakash Raichur, Hans-Christoph Schneider, Daniel Crowther, Corrado Priami

**Affiliations:** 1 The Microsoft Research - University of Trento Centre for Computational and Systems Biology, Rovereto, Italy; 2 Department of Mathematics, University of Trento, Italy; 3 Insulin Biology and Counter Regulatory Hormones Dept. R&D Diabetes Division,Sanofi-Aventis Deutschland GmbH, 65926 Frankfurt am Main, Germany; 4 R&D TMED Translational Informatics, Sanofi-Aventis Deutschland GmbH, 65926 Frankfurt am Main, Germany

## Abstract

Recent research adds to a growing body of literature on the essential role of ceramides in glucose homeostasis and insulin signaling, while the mechanistic interplay between various components of ceramide metabolism remains to be quantified. We present an extended model of C16:0 ceramide production through both the *de novo* synthesis and the salvage pathways. We verify our model with a combination of published models and independent experimental data. In silico experiments of the behavior of ceramide and related bioactive lipids in accordance with the observed transcriptomic changes in obese/diabetic murine macrophages at 5 and 16 weeks support the observation of insulin resistance only at the later phase. Our analysis suggests the pivotal role of ceramide synthase, serine palmitoyltransferase and dihydroceramide desaturase involved in the *de novo* synthesis and the salvage pathways in influencing insulin resistance versus its regulation.

Ceramides (Cer) are a family of lipid molecules that play an active role in glucose homeostasis, insulin signaling and, ultimately, the diabetic phenotype^1^,^2^. Two primary pathways through which ceramides are produced in the cell are the condensation of palmitate and serine (called *de novo* synthesis) and re-acylation of sphingosine (salvage pathway). In both cases, ceramide (dihydroceramide, in the case of the de novo synthesis pathway) is produced by ceramide synthase (CERS) through N-acylation of a sphingoid base. Mammalian CERS occurs in 6 isoforms (CERS1-6) with differing binding preference for specific fatty acid chain lengths. CERS6, in particular, is specific to C14 and C16 acyl chain lengths, and has been associated with obesity and insulin resistance^3^. The primary mechanism through which ceramide promotes insulin resistance is by inhibiting the activity of Akt/PKB, which is an essential facilitator of glucose transport into the cell. Ceramide blocks the activity of Akt/PKB by two independent mechanisms, i.e., by stimulation of Akt dephosphorylation via protein phosphatase 2A (PP2A) and blocking the translocation of Akt via PKC*ζ*^4^. Ceramide activates PP2A, which inhibits the action of Akt/PKB by impairing Akt serine phosphorylation. The result of this inhibition is decreased translocation of glucose transporter type 4 (GLUT4) to the plasma membrane and hence decreased uptake of glucose. In this study, we extended the dynamic model of the *de novo* synthesis of C16:0 ceramide (from here on we omit the C16:0 notation) in ref. 5 (Supplementary Table S1) with the salvage pathway (Supplementary Table S2). The deterministic extension of the model in ref. 5 is used to tune a stochastic version of the same model implemented in *ℓ*: a stochastic imperative, domain specific language^6^,^7^. The quantitative parameters of our model are extracted from lipidomic data on RAW264.7 cells^8^ (a mouse leukemic macrophage cell line) and validated on primary macrophages^9^ (bone marrow derived macrophages, BMDM). The cells were treated with the pro-inflammatory compound Kdo(2)-lipid A (KLA). RAW264.7 cells experiment was assessed at 0, 0.5, 1, 2, 4, 8, 12, and 24 hrs, whereas BMDM experiment at 0, 0.25, 0.5, 1, 2, 4, 8 and 20 hrs. Following^5^, we assume that there are diacylglycerol (DAG)- and phosphatidylcholine (PC)- mediated reactions that transform dihydroceramide to dihydrosphingomyelin and vice versa. These reactions are analogous to the reactions involving ceramide and sphingomyelin, which connect ceramide *de novo* synthesis with the sphingomyelinase pathway. We simulated our model both deterministically and stochastically to account for low abundances of metabolites. The outcome of the simulations predicts the trend of sphingolipid accumulation in CERS6 knockout mice^3^ as well as the insulin resistance aetiology in *ob/ob* mice^10^. Finally, we performed a sensitivity analysis to identify the key enzymes and reactions that regulate sphingolipid metabolism.

## Results

Working with an extended model of the one presented by Gupta *et al*.^5^ to include the interplay between ceramide and sphingosine, the main result is the ability of our model to explain mechanistically the interplay between sphingolipid metabolism, specifically ceramide, and insulin resistance. We experimented on our model by focusing on two cases: (i) the availability of CERS6, and (ii) the groups of enzymes that are identified as significantly differentially-expressed in obese mice. The data on obese mice is from isolated adipose tissue macrophages from 5 and 16 week *ob/ob* (i.e., genetically obese) and wild type C57BL/6 mice, both fed standard chow diets (for detailed study methods, see ref. 10). We then performed a sensitivity analysis of the model.

### CerS6 availability

We investigated the response to variation in CERS6 fold change (FC), as this enzyme plays a central role in the *de novo* production of (primarily C16:0) ceramide, catalyzing dihydroceramide (dhCer) starting from sphinganine, and in the salvage production, recycling ceramide from sphingosine. A substantial reduction of CERS6, for example, as a result of the effects of drugs such as fumosin B1, has the effect of blocking both *de novo* and salvage pathways, leaving only the ceramide production that occurs by sphingomyelinase. Moreover, as shown in ref. 3, among all CERS enzymes, only CERS6 adipose tissue expression is significantly correlated with BMI, hyperglycemia and glucose infusion rate in human subjects.

**Figure 1:**
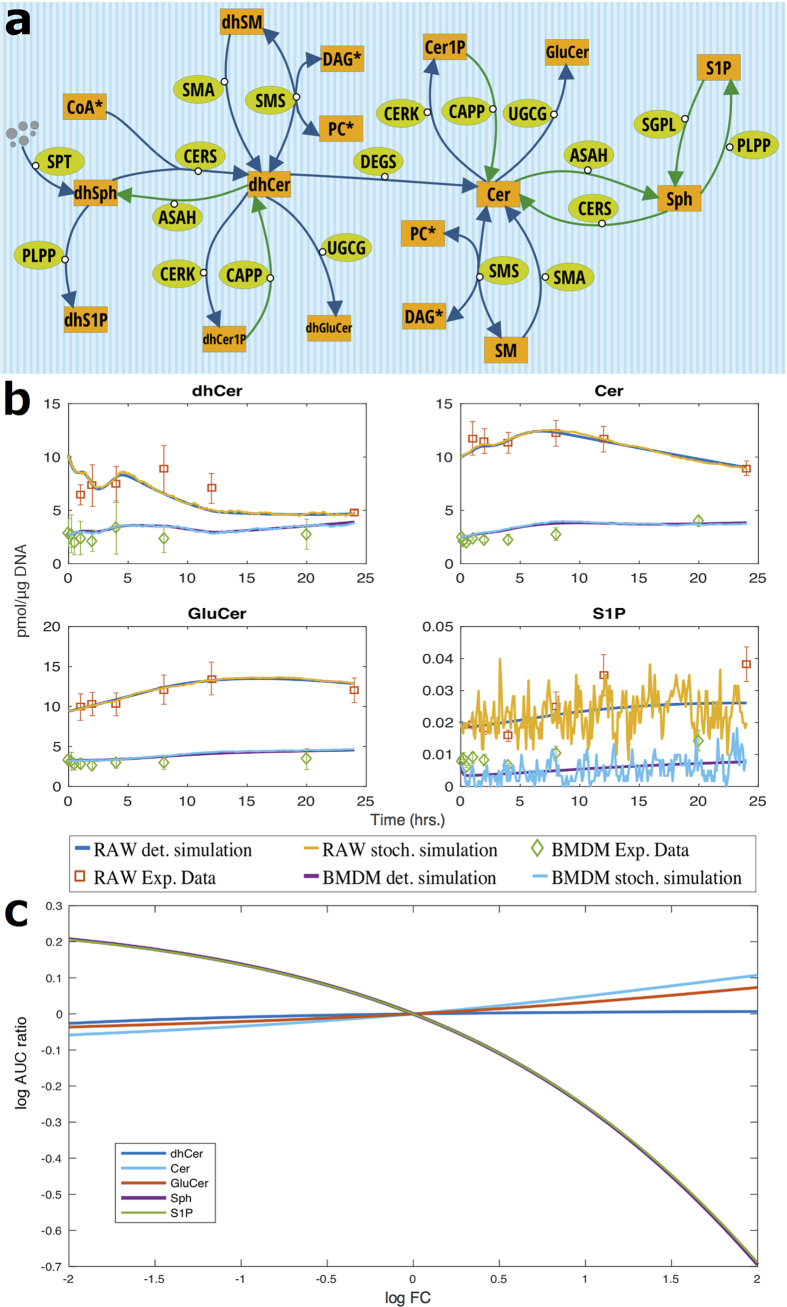
(**a**) The model extends the one in ref. 5 with additional reactions identified in the literature in green. Degradation and production reactions are omitted. Metabolites with time-dependent variables are marked with the symbol ‘*’. (**b**) Simulation results of the extended models, both deterministic and stochastic, for the concentrations in RAW 264.7 cells and BMDM measured in pmol/*μ*g DNA. The model simulates all the sphingolipids involved in the ceramide pathway. For the stochastic simulations a scaling factor 1000 is used. *x-axis*: time in hours. (**c**) The log_2_ AUC-FC/AUC-Control ratio for the sphingolipids in the legend. The fold-change is varied from 0.25 to 4.

Our model provides a mechanistic explanation of the results of ref. 3: the contribution of CERS5 in ceramide synthesis in macrophages is three orders of magnitude smaller than the one of CERS6. As a consequence, the extended model includes a reaction that merges the effect of CERS5 and CERS6. In our analysis, in agreement with^5^, we thus consider only the FC of CERS6, as it is the main contributor for the dynamics in the sphingolipid pathway, and FC of CERS5 remains negligible in comparison. Figure 1 shows results of the simulation, in particular with (Fig. 1c) showing that decreasing CERS6 results in an decrease in ceramide as well as an increase in sphingosine-1-phosphate.

### Differentially expressed enzymes in *ob/ob* mice

While CERS6 plays a known role in the diabetic phenotype, dysregulation of biological systems is often the result of altered behavior in many interacting components. Therefore, we focused our analysis on multiple enzymes that were found to be differentially expressed in macrophages of *ob/ob* and wild type mice.

**Figure 2:**
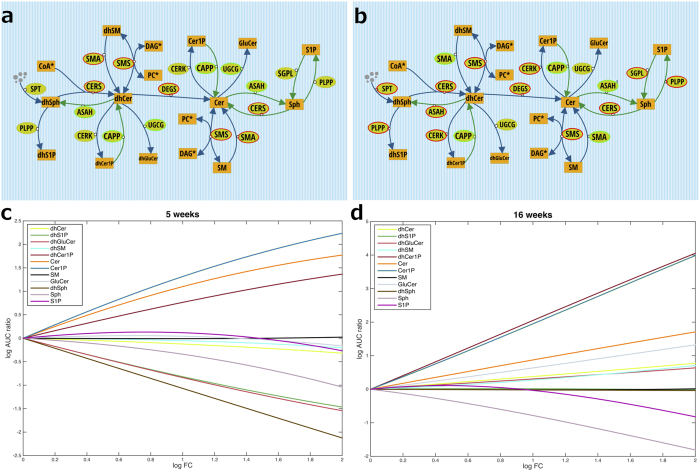
(**a**,**b**) The up (red boundary) and down (green boundary) regulated enzymes in obese mice, according to genes significantly differentially expressed, after 5 (Supplementary Table S3) and 16 weeks (Supplementary Table S4) of chow diet. (**c**,**d**) the log_2_ AUC-FC/AUC-Control ratio for the sphingolipids in the legend. The fold-change of the enzymes highlighted in (**a**,**b**) are varied from 1 to 4.

Results from simulations suggest that sphingolipid metabolism in the obese mouse is affected after 5 weeks (Fig. 2c). However, the sphingolipids related to insulin action, ceramide, glucosylcermide (GluCer) and S1P are balanced: GluCer and S1P, are either stable or decreasing, ceramide increases and the mechanisms of insulin resistance due to Akt activity remain unaffected. These observations are in agreement with^10^: after 5 weeks, ob/ob mice show signs of early insulin resistance, compared with wild-type mice, however show well-controlled glycemia. Moreover, the model indicates that the affected sphingolipid metabolism maintains a balance between sphingolipids involved in insulin signaling.

Simulations suggest that *ob/ob* mice metabolism is highly affected after 16 weeks with a general up-regulation of sphingolipids, including the ones involved in insulin signaling (Fig. 2d). This suggests potential impairment of insulin signaling and the development of insulin resistance and glucose intolerance. The model indicates an impairment of the ratio between the sphingolipids involved in insulin signaling. In agreement with Prieur *et al*.^10^, this can be the cause of the obesity-induced insulin resistance^11^,^12^, which is stronger at 16 weeks than at 5 weeks, leading to severe insulin resistance and glucose intolerance.

### Sensitivity analysis

**Figure 3:**
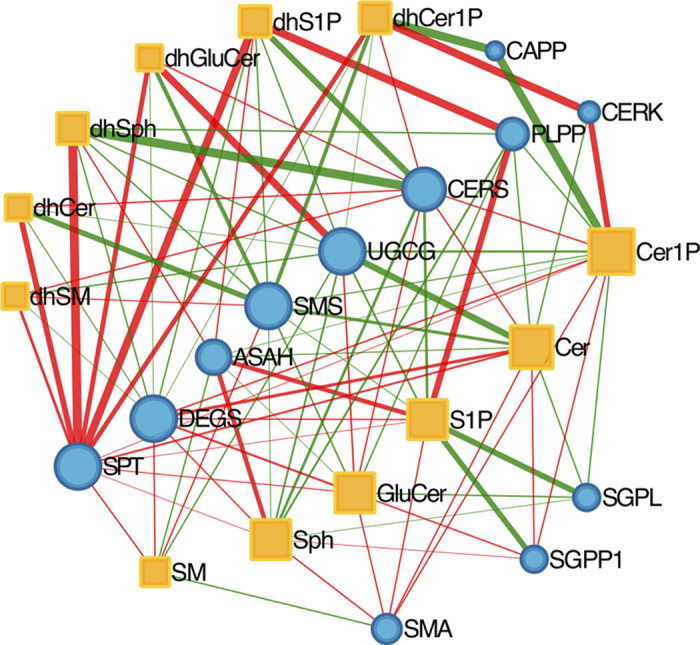
The network of interactions obtained from the sensitivity analysis for enzymes. Edges are red if the increase of the rate causes an increase of the concentration of the sphingolipid node; green if the concentration decreases. The thickness of the edges is proportional to the log_2_ of the AUC ratio. Orange rectangles and blue circles, respectively, are the sphingolipids and the enzymes.

We performed parametric sensitivity analysis to test the model and to highlight the key reactions and enzymes for the behavior of the system and estimate the effect of each rate or enzyme on the concentration of each sphingolipid. Figure 3 illustrates the results of sensitivity analysis as a network, with the width of edges indicating the strength of effect of enzymes on metabolite abundance. Our results demonstrate that while the concentrations of enzymes like ceramide-activated protein phosphatase (CAPP), ceramide kinase (CERK), sphingosine-1-phosphate lyase (SGPL1) and sphingosine-1-phosphate phosphatase (SGPP1) have strong effect on specific sphingolipids, other enzymes like CERS, ceramide glucosyltransferase (UGCG), dihydroceramide desaturase (DEGS), sphingomyelin synthase (SMS), ceramidase (ASAH), sphingomyelinase (SMA) and serine palmitoyltransferase (SPT) have a more diffuse effect throughout the model.

## Discussion

We used a combination of deterministic and stochastic simulations to provide a dynamic account of the mechanistic processes of sphingolipid metabolism. We extended and refined the model by Gupta *et al*.^5^ to include the interplay between ceramide and sphingosine. We used our model to test different conditions for CERS6 availability and various combinations of enzymes that are differentially expressed in *ob/ob* mice after 5 and 16 weeks. We quantified the effect of each single enzyme in the pathway, through sensitivity analysis, identifying the main regulators of sphingolipid production.

The data used to identify the parameters of the model are taken from cell cultures, where fast and slow metabolic interactions co-occur, and this is a source of intrinsic noise; stochastic simulations are useful in capturing the fluctuations due to these variations in reaction rates. Moreover, the measurements of metabolite concentrations in this system vary in orders of magnitude. Stochastic simulations are instrumental for capturing the noise that emerges in experimental observations^13^ (see Supplementary Fig. S4). Figure 1b shows that the stochastic simulations are closer to experimental observations than deterministic simulations on both RAW 264.7 and BMDM cells. Deterministic simulations, on the other hand, are better for sensitivity analysis and monitoring the average behavior.

We then extended the model to analyze the cross-talk between ceramide *de novo* synthesis and the salvage pathway, where ceramide is produced by recycling sphingosine^14^ (Fig. 1a). Sphingosine is involved in Cer synthesis inside lysosomes and mitochondria, and sphingosine-1-phosphate (S1P) plays a central role in insulin signaling and inflammatory response^15^. By including sphingosine and S1P, the extended model is a comprehensive exposure of the processes that link ceramide metabolism to the diabetic phenotype. Moreover, the extended model takes the role played by ceramide-1-phosphate phosphatase (CAPP) into account, which produces ceramide from ceramide-1-phosphate^16^.

Our simulations showed that the decrease of CERS6 results in a decrease of ceramide, as expected, as well as an increase in sphingosine-1-phosphate (Fig. 1c). Sphingosine-1-phosphate (S1P) can be reversibly produced from ceramide via sphingosine, and plays a well-studied role in insulin signalling. Recent work by Mullen *et al*.^17^ demonstrated that combined knockdown of CERS2, CERS5 and CERS6 resulted in elevated levels of S1P in an adenocarcinoma cell line. The interplay between the levels of ceramide and sphingosine-1-phosphate (S1P) plays a role in the control of the Akt pathway, which in turn influences insulin action as well as the fate of the cell^18^,^19^,^20^. This suggests that, for macrophage sphingolipid metabolism, the balance of these two sphingolipids may explain why even with high-fat diet, CERS6-knockout mice did not show significant differences in insulin action and glucose tolerance in comparison with wild type high-fat diet-fed mice^3^. Conversely, as CERS6 abundance increases, the simulations suggest that the increase of both ceramide and glucosylceramide (GluCer) affects the Akt/PKB insulin signaling pathway, which is correlated with increased CERS6 expression.

In ref. 5 the abundances of the four metabolites DAG, phosphatidylcholine, sphinganine and palmitoyl-CoA are modeled with time-dependent variables obtained as linear interpolations of the experimental data. This approach is based on the physiological observation that KLA treatment primarily affects these four components, and the treatment induced variation of concentrations is enough to capture the effect of the treatment for most of the metabolites. Our extended model refines the representation of *de novo* synthesis by replacing the time-dependent functions for sphinganine with mechanistic components and, as a consequence, provides a characterization of the underlying biochemical processes. We aggregated the reactions between same metabolites that are mediated by different enzymes, thereby assessing of the aggregated influence of each sphingolipid over other sphingolipids without compromising accuracy. We initially assumed that the enzyme levels remain constant during the experiments, as in ref. 5. Furthermore, we successfully tested the consistency of the results in the presence of significant perturbations on enzyme concentrations (Supplementary Fig. S8). We were able to significantly reduce the number of rates to fit without affecting the precision of the model, and avoid compensation effects of parallel reactions. The stochastic simulations are performed using *ℓ*, a domain-specific modeling language. The results of the deterministic and stochastic simulations compared with experimental data for RAW 264.7 and BMDM cells are in Fig. 1.

The sensitivity analysis of the enzymes highlighted a strong role for SPT, which showed positive effects on the abundance of a range of metabolites. The reaction carried out by SPT - the condensation of serine and palmitoyl CoA to produce dihydrosphingosine (dhSph) - has been shown to be a rate-limiting step in de novo sphingolipid biosynthesis^21^. Humans possess three variants of SPT (SPTLC1, SPTLC2, SPTLC3), and SNPs in all three have been found to be significantly associated with type 2 diabetes and related phenotypes^22^,^23^,^24^,^25^,^26^,^27^,^28^. Likewise, treatment with myriocin (a specific inhibitor of SPT) substantially reduces ceramide synthesis and ameliorates insulin resistance in diabetic rodents^29^,^30^.

The sensitivity analysis also highlights the diffused effect of DEGS, the enzyme that catalyzes the transformation of dhCer into ceramide, and connects the dh and non-dh parts of the pathway. A significant reduction of this enzyme removes the dhCer contribution from ceramide production, and an increase in DEGS promotes ceramide production. Recent work has shown that signaling targets of ceramide are not affected by similar levels of dhCer, which suggests that the enzyme DEGS is essential in cell regulation^31^ and plays a role on glucose homeostasis as well: multiple SNPs in DEGS are significantly associated with 2 hour glucose, mice lacking DEGS are resistant to dexamethasone-induced insulin resistance and DEGS-knockdown mice myoblast are protected from palmitate-induced ceramide-mediated insulin resistance^22^,^28^,^29^,^32^.

Collectively, our results illustrate patterns in sphingolipid metabolism that mechanistically link ceramides and related bioactive lipids to insulin resistance. By perturbing CERS6 we observed changes in sphingolipid abundances that are consistent with improvement in insulin signalling, however further work would be required to assess how this may affect whole-body glucose homeostasis. Furthermore, in agreement with recent studies in rodents and humans, sensitivity analysis of our model highlighted a strong functional role of SPT and DEGS in regulating abundance of multiple sphingolipid metabolites that are fundamentally linked to metabolic health.

## Methods

We implemented two versions of the model for deterministic and stochastic simulations. Because both of these implementations are based on mass action kinetics, they can be considered as two equivalent implementations of the same model. The stochastic simulations capture some fluctuations that are not captured by the deterministic simulations. The deterministic simulations, on the other hand, are more efficient and enable fitting procedures.

### Deterministic implementation

We implemented the deterministic version of the model using ordinary differential equations (ODE) in Matlab, and we used the built-in ODE solver functions ode23 and ode45. Sometimes we used a lower order Runge-Kutta method to speed up the simulation, and compared the results with higher order methods to quantify the error. Time dependent variables are included as in ref. 5; they are encoded as linear interpolation of the related experimental concentrations.

### Stochastic implementation

We implemented the time-continuous discrete stochastic version of the model using the domain specific *ℓ* language designed to model chemical reactions and biological systems^6^,^7^. *ℓ* is equipped with a built-in stochastic simulation engine, based on Gillespie algorithm^33^. The mass action kinetics allowed us to use the deterministic rates also for the stochastic implementation by using conversion factors, see, e.g. ref. 7. The number of molecules required for the stochastic simulation are thus obtained by using the transformation from the concentration *pmol*/*μg* of DNA to number of molecules, that is, by multiplying with the expression *AvogadroNumber* · 10^−12^ · 10^−6^/*ScalingFactor*. We performed simulations with different stochastic seeds and scaling factors that emphasize the stochastic noise, and we compared these results with the experimental data. The yellow plots in Fig. 1b and Supplementary Fig. S4 are obtained with a scaling factor 1000. Time dependent variables were included in the propensity calculation of the reactions, and their amounts are determined as linear interpolation of the related experimental number of molecules. For example, the propensity function at time *t* of 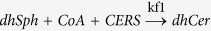, is


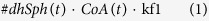


where *#dhSph(t*) is the simulated number of molecules of *dhSph* at time 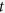, kf1 is the stochastic rate constant, and *CoA(t*) is the time dependent function that accounts for the number of *CoA* molecules at time *t*.

### Enzyme concentrations

The concentrations of the enzymes in the model are initially kept constant during the duration of the experiments as in ref. 5, and their amounts are calculated by parameter estimation. For example, the estimated parameter kf12 of 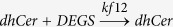 is [*DEGS*] · *kf*12′, where *kf*12′ is the actual kinetics value and [*DEGS*] is the enzyme concentration.

### Enzyme Availability

To validate the model results with respect to the variations in the availability of the enzymes, we have quantified the effect of reducing each enzyme on each metabolite. To measure the accumulated effect of these changes on the metabolites, we used the Area Under the Curve (AUC) of the simulated time-series. We used the log_2_ of the AUC ratio for the case with reduced enzyme availability and the AUC for the control case, that is, log_2_(AUC-Reduced/AUC-Control). This allowed us to quantify and compare the variations in AUC, depicted as heatmaps. We performed this by scaling the reaction rate constants that are mediated by the selected enzyme from 0.1 to 1. These results are depicted in Supplementary Fig. S6.

To further assess the robustness of the model, we dynamically perturbed the concentration of the enzymes. We first considered the perturbations at random time points. Following this, we applied perturbations at fixed time points in order to compare the two behaviors, and verify that they are in agreement. For this, at each time point, we considered random normally distributed fold changes (FC) for all the enzymes. These fold changes are included in the rate constant as a factor of 2^FC^. For the perturbations, we tested a variety of time points and standard deviations from 6 to 240 and from 0.1 to 1, respectively. Supplementary Figs S7 and S8 depict the results for 10000 different simulations with a standard deviation of 0.5. As expected, varying the standard deviations result in proportional variations of the outputs. Supplementary Fig. S6 shows that the output of the model is consistent with the dynamics we have considered as control, also in the presence of significant perturbations on enzyme concentrations (Supplementary Figs S7 and S8). The dynamics of all the sphingolipids in our conclusions in the main text show good agreement with the experimental data (Supplementary Figs S7 and S8).

### Parameter Estimation

The extended model includes 29 reactions, with unknown rates. We carried out a deterministic parameter estimation procedure, based on *non-linear least squares method*. To take the differences in concentration of the sphingolipids into account and to ensure that the fitting procedure is not used in a biased manner by the abundance of any sphingolipid, we used a weighted objective function, where for each sphingolipid and each time point we considered the squared-relative-error. The objective function that we minimized is


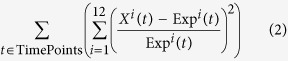


where *X*^*i*^(*t*) and Exp^*i*^(*t*) are the simulated and experimental values for the i-th, non-time dependent, sphingolipid at the time point *t*, and TimePoints is the set of the experimental measurements at time *t* ∈ {0.5, 1, 2, 4, 8, 12, 24} hours.

We minimized this expression using the *lsqnonlin* Matlab function (trust-region-reflective algorithm) by using a multi-start approach: the algorithm starts from a number of randomly generated starting points (our case 200) within the feasible regions and from each point it executes the *lsqnonlin* function. This procedure for parameter estimation provided excellent fitting results (Fig. 1b and Supplementary Fig. S4).

### Validation

Since the BMDM dataset does not include all the data, for the time dependent variables (Acyl-CoA 16, DAG and PC) we have used data from RAW 264.7 cells. The results of this simulation are depicted in Supplementary Fig. S5. The model, tested on the new data, correctly reproduces the behavior of most of the metabolites, in particular these metabolites that are involved in our conclusions. However, the dynamics of dhSph is not correctly captured by the model. Our investigation on this highlights the role of the reaction that synthesizes dhSph (reaction 22). Since the concentration of dhSph is much smaller in the BMDM data than in RAW 264.7 cells, we varied its rate accordingly. As a consequence, the model results are closer to experimental data.

### Microarray analysis

A microarray dataset (accession number GSE36669) was obtained from Gene Expression Omnibus, representing isolated adipose tissue macrophages from 5 and 16 week *ob/ob* and wild type C57BL6 mice, both fed standard chow diets (for detailed study methods, see ref. 10). Data were normalized using the *rma* function from the oligo R library, then filtered to remove probes in the lowest 10% expression and lowest 10% variance. Data were then analyzed using the limma library, to identify probes that were significantly differentially expressed between *ob/ob* and wild type mice at 5 and 16 weeks. P values were corrected for multiple testing using the Benjamini & Hochberg method^34^.

### Fold change experiments

In order to test how the model responds to the variations in the concentration of the enzymes that emerge in conditions related to obesity, we modeled the variation of the amount of certain enzymes in terms of their fold change (FC) with respect to the experimental microarray data. To cluster the enzymes we identified two sets of genes: those contained in our model and differentially expressed at 5 weeks (Supplementary Table S3) those contained in our model and differentially expressed at 16 weeks (Supplementary Table S4). In the FC experiments, we included the variation in fold change in the model as the product of [*DEGS*] · *FC* · kf12′, thereby assuming that the variations in the fold change of gene expression affect the concentrations of the related enzymes, and this affects the propensity of the reactions that involve these enzymes. In particular, if the enzyme is overexpressed FC is given as 2^*x*^, if it is underexpressed as 2^−*x*^, where *x* is the amount of change we simulated. To measure the accumulated effect of these changes, in accordance with the experimental data, we have considered the log_2_ of the ratio of AUC for the case with varied fold change of the enzymes and the AUC of the control case where FC = 1, that is, log_2_(AUC-FC/AUC-Control). This provides an estimate of the cumulative effect over the whole experiment with respect to the varied fold change of the enzyme. Logarithm is used to highlight the cases in which the fold change has a decreasing effect on the accumulated effect of the metabolite.

### Compartmentalization

Sphingolipid metabolism is a complex system that takes place in different parts of the cell, from the ER to the cell membrane^18^,^35^. Regarding the specific compartmentalization of the metabolites, to the best of our knowledge, there is no data available that is suitable for modeling. Other approaches have been tested in the literature, such as in ref. 36, where the authors proposed a model for sphingolipid metabolism that includes compartments. However, their results are neither compared with experimental data, nor quantitatively justified. Moreover, the model in ref. 36, despite the lack of experimental verification, includes more than 120 free parameters, which introduces additional challenges in terms of interpretation of the results. In contrast, our model includes only 29 parameters, which are instantiated by using experimental data, and verified by independent data and sensitivity analysis.

We have, however, addressed the compartmentalization of certain components within the realm of our model. We tested our model in order to quantify how the effect of impairment on the transport mechanisms^18^ would affect its output. To this end, we analyzed the sphingolipids that may be more affected by the transport impairment. In particular, we tested our results by varying the initial concentrations of the metabolites that are known to be subject to transport between compartments by a factor from 1 to 0.2. These results are depicted in Supplementary Fig. S9. These simulations indicate that our results are not vulnerable to perturbations in the availability of sphingolipids due to alteration in transport.

### Sensitivity Analysis

We performed a parametric sensitivity analysis, considering for each reaction the estimated rate and varying it under mass action law. We considered 4 orders of magnitude fold change interval starting from 0.01 up to 100 that covers possible metabolic perturbations of the system. The parameter fold changes are included in the model in the same way as for the enzyme fold changes. We ran simulations by varying these fold change values, and we measured the impact of these changes to the system in terms of AUC ratio for each sphingolipid. We performed the same kind of analysis for enzymes, with varying FC from 0.01 up to 10.

This data is used to produce a network of interactions. We used orange rectangular nodes to represent the sphingolipids and blue circles to represent the rates or the enzymes. The dimension of the nodes is proportional to the number of incident edges. We used color edges to differentiate the effect of rates on sphingolipids: an edge is red if the rate increase causes concentration of the lipid increase; it is green if the concentration decreases. Undirected edges are chosen for increasing readability, however they are directed from the rates or the enzymes to the sphingolipids (Fig. 3 and Supplementary Fig. S1).

We weighted the edges of the network proportionally to the base two logarithm of the AUC ratio. Therefore an edge has the same width if the effect on a sphingolipid is doubling or halving its concentration. In particular, we determined the value to use for this representation with respect to the results of the sensitivity analysis. We identified a value in the range where the concentrations vary in a monotone way according to FC. In this case, we choose a fold change of 4. To improve the readability of the network we represented only the interaction such that |AUC ratio −1| > 0.01. We produced in the same way the interaction network for the enzymes and chose the interactions such that |AUC ratio −1| > 0.001.

The networks are obtained from simulation data, and processed using the igraph R library^37^. Finally the network figure was produced using Cytoscape, and the organic layout algorithm was used to improve the readability.

## Additional Information

**How to cite this article**: Reali, F. *et al*. Mechanistic interplay between ceramide and insulin resistance. *Sci. Rep.* **7**, 41231; doi: 10.1038/srep41231 (2017).

**Publisher's note:** Springer Nature remains neutral with regard to jurisdictional claims in published maps and institutional affiliations.

## Supplementary Material

Supplementary File 1
