# Supplementary material for: Mechanistic interplay between ceramide and insulin resistance
Source: Sci Rep. 2017 Jan 23;7:41231. doi: 10.1038/srep41231 (PMC5253739; doi:10.1038/srep41231)
Supplement: Supplementary File 1 [file srep41231-s1.pdf]

Supplementary Material
Mechanistic interplay between ceramide and insulin resistance

Federico Reali[+1,2], Melissa J. Morine[+1], Ozan Kahramanoğulları[+1,2], Suryaprakash Raichur[3], Hans-Christoph Schneider[3], Daniel Crowther[3], and Corrado Priami[*1,2]

[1]The Microsoft Research - University of Trento Centre for Computational and Systems Biology, Rovereto, Italy, {morine,reali,ozan,priami}@cosbi.eu
[2]Department of Mathematics, University of Trento, Italy
[3]Sanofi, Frankfurt, Germany, {Daniel.Crowther, Hans-Christoph.Schneider, Surya.Prakash2}@sanofi.com
[*]corresponding.author@email.example
[+]these authors contributed equally to this work

Supplementary Material

Reaction N.	Reaction	Rate Identifier	Deterministic Rate	Stochastic Rate
1	dhSph + CoA16 + CERS6 $\rightarrow$ dhCer	kf1	13	2.158751e-05 $\cdot$ sf
2	dhSph + CoA16 $\rightarrow$ dhCer	kf2	5.940000e-02	9.863833e-08 $\cdot$ sf
3	dhCer $\rightarrow$	kf3	1.060000e-03	1.060000e-03
4	dhSph + Sphk1 $\rightarrow$ dhSph1P	kf4	3.740000e-04	3.740000e-04
5	dhSph + Sphk2 $\rightarrow$ dhSph1P	kf5	1.570000e-02	1.570000e-02
6	dhSph1P $\rightarrow$	kf6	5.200000e-01	5.200000e-01
7	dhCer + UGCG $\rightarrow$ dhGlcCer	kf7	2.020000e-02	2.020000e-02
8	dhGlcCer $\rightarrow$	kf8	2.900000e-01	2.900000e-01
9	dhCer + Sms1 + PC $\longleftrightarrow$	kf9	1.160000e-01	1.926270e-07 $\cdot$ sf
	dhSM + Sms1 + DAG	kb9	3.900000e-01	2.856360e-06 $\cdot$ sf
10	dhCer + Sms2 + PC $\longleftrightarrow$	kf10	1.720100e+00	6.476254e-07 $\cdot$ sf
	dhSM + Sms2 + DAG	kb10	1.080000e-01	1.429758e-06 $\cdot$ sf
11	dhSM + SMPD1 $\rightarrow$ dhCer	kf11	8.610000e-01	1.080000e-01
12	dhSM $\rightarrow$	kf12	3.000000e-02	3.000000e-02
13	dhCer + CERK $\rightarrow$ dhCerP	kf13	1.180000e-04	1.180000e-04
14	dhCerP $\rightarrow$	kf14	1.660000e-01	1.660000e-01
15	dhCer + DEGS1 $\rightarrow$ Cer	kf15	9.250000e-03	9.250000e-03
16	dhCer $\rightarrow$ Cer	kf16	8.000000e-01	8.000000e-01
17	Cer + CERK $\rightarrow$ CerP	kf17	1.180000e-04	1.180000e-04
18	CerP $\rightarrow$	kf18	1.130000e-01	1.130000e-01
19	Cer + Sms1 + PC $\longleftrightarrow$	kf19	1.160000e-01	1.926270e-07 $\cdot$ sf
	SM + Sms1 + DAG	kb19	3.900000e-01	9.282630e-08 $\cdot$ sf
20	Cer + Sms2 + PC $\longleftrightarrow$	kf20	5.590000e-02	6.476254e-07 $\cdot$ sf
	SM + Sms2 + DAG	kb20	1.670000e-03	2.236632e-06 $\cdot$ sf
21	SM + SMPD1 $\rightarrow$ Cer	kf21	1.346900e+00	1.670000e-03
22	SM $\rightarrow$	kf22	1.060000e-02	1.060000e-02
23	Cer $\rightarrow$	kf23	0	0
24	Cer + UGCG $\rightarrow$ GlcCer	kf24	2.020000e-02	2.020000e-02
25	GlcCer $\rightarrow$	kf25	3.170000e-03	3.170000e-03

Table S1. Reactions and stochastic rates obtained from the deterministic rates in[1]. *sf* refers to the scaling factor.

Reaction N.	Reaction	Rate Identifier	Deterministic Rate	Stochastic Rate
1	dhSph + CoA + CERS $\longrightarrow$ dhCer	kf1	1.2523e+01	2.0794e-05 · sf
2	dhCer $\longrightarrow$	kf2	5.3996e-01	5.3996e-01
3	dhSph + PLPP $\longrightarrow$ dhS1P	kf3	3.5455e-03	3.5455e-03
4	dhS1P $\longrightarrow$	kf4	1.5116e-01	1.5116e-01
5	dhCer + UGCG $\longrightarrow$ dhGluCer	kf5	6.8664e-02	6.8664e-02
6	dhGluCer $\longrightarrow$	kf6	3.2676e-01	3.2676e-01
7	dhCer + SMS + PC $\longleftrightarrow$	kf7	9.2076e-02	1.5290e-07 · sf
	dhSM + SMS + DAG	kf28	4.7810e-01	7.9390e-07 · sf
8	dhSM + SMA $\longrightarrow$ dhCer	kf8	2.0220e-08	2.0220e-08
9	dhSM $\longrightarrow$	kf9	4.1706e-02	4.1706e-02
10	dhCer + CERK $\longrightarrow$ dhCer1P	kf10	7.6142e-04	7.6142e-04
11	dhCer1P + CAPP $\longrightarrow$ dhCer	kf11	4.0986e-01	4.0986e-01
12	dhCer + DEGS $\longrightarrow$ Cer	kf12	1.3904e-01	1.3904e-01
13	Cer + CERK $\longrightarrow$ Cer1P	kf13	2.9955e-04	2.9955e-04
14	Cer1P + CAPP $\longrightarrow$ Cer	kf14	1.7897e-01	1.7897e-01
15	Cer + SMS + PC $\longleftrightarrow$	kf15	2.3484e-03	3.8996e-09 · sf
	SM + SMS + DAG	kf29	2.0752e-02	3.4460e-08 · sf
16	SM + SMA $\longrightarrow$ Cer	kf16	9.6067e-05	9.6067e-05
17	SM $\longrightarrow$	kf17	6.4391e-05	6.4391e-05
18	Cer $\longrightarrow$	kf18	1.0800e-02	1.0800e-02
19	Cer + UGCG $\longrightarrow$ GluCer	kf19	1.2546e-01	1.2546e-01
20	GluCer $\longrightarrow$	kf20	9.8926e-02	9.8926e-02
21	dhCer + ASAH $\longrightarrow$ dhSph	kf21	1.3685e-02	1.3685e-02
22	null + SPT $\longrightarrow$ dhSph	kf22	4.6336e+00	(2.7904e+06)/ sf
23	Cer + ASAH $\longrightarrow$ Sph	kf23	2.6853e-02	2.6853e-02
24	Sph + CERS $\longrightarrow$ Cer	kf24	2.4217e-02	2.4217e-02
25	Sph + SK $\longrightarrow$ S1P	kf25	3.7867e-02	3.7867e-02
26	S1P + SGPP1 $\longrightarrow$ Sph	kf26	4.9200e+00	4.9200e+00
27	S1P + SGPL $\longrightarrow$	kf27	3.7856e+00	3.7856e+00

Table S2. Reactions of our extended model.

logFC	adj.P.	symbol	aggregated symbol	$AveExpr_{WT}$	$AveExpr_{ob/ob}$
−0.452620099	0.008182273	Sgpp1	SGPP1	9.221666078	8.769045979
−0.224976984	0.091131914	Asah2	ASAH	7.750207047	7.525230062
−0.216372605	0.096486253	Asah1	ASAH	10.39519443	10.17882183
−0.180378296	0.09595853	Ugcg	UGCG	8.181176815	8.000798519
0.497378687	0.003163028	Degs1	DEGS	8.628122177	9.125500863
0.454196511	0.016684256	Smpd3	SAM	6.563311134	7.017507645
0.346984067	0.011837382	Sgms1	SMS	8.109475589	8.456459656
0.970942051	0.008430373	Sgms2	SMS	5.871574472	6.842516523
0.510300826	0.003532425	Cers6	CERS	8.087639879	8.597940705

Table S3. Statistical analysis results for genes contained in our model. Differential expression values refer to the difference between wildtype (WT) and *ob/ob* mice at 5 weeks.

logFC	adj.P.	symbol	aggregated symbol	AveExpr_{WT}	$\text{AveExpr}_{ob/ob}$
0.251283323	0.009757826	Asah1	ASAH, ASAH[1]	11.32289211	11.57417544
−0.400699156	0.000899179	Asah2	ASAH, ASAH[1]	8.618283842	8.217584686
−0.19660911	0.078807427	Acer1	ASAH[1]	5.177626666	4.981017556
−0.625195789	7.90e-05	Acer2	ASAH[1]	7.548612508	6.923416719
1.038252808	1.09e-07	Acer3	ASAH	7.721198262	8.75945107
0.588336958	1.34e-05	Cerk	CERK	8.527943995	9.116280954
0.60453069	7.26e-06	Degs1	DEGS	9.737870426	10.34240112
−1.883316456	1.12e-08	Ppap2b	PLPP	11.79434036	9.911023905
0.336052217	0.020480217	Ppap2c	PLPP	8.463706403	8.799758619
0.343115755	0.001608825	Sptlc1	SPT	9.044525454	9.38764121
−0.198809527	0.072928862	Smpd3	SMA	7.912221158	7.713411631
0.470062309	3.91e-05	Sgms1	SMS	8.633440478	9.103502786
0.182996148	0.05350511	Sphk2	PLPP	7.777322825	7.960318974
0.812523823	5.45e-07	Sgpl1	SGPL	10.93098122	11.74350504
0.947997388	1.04e-07	Cers6	CERS	8.718705273	9.666702661
0.206366580	0.025389854	Cers5	CERS	8.868672068	9.075038647

Table S4. Statistical analysis results for genes contained in our model. Differential expression values refer to the difference between wildtype (WT) and *ob/ob* mice at 16 weeks. ASAH is the name that summarize enzymes that mediate reaction 21, ASAH[1] those which mediate reaction 23.

References

1. Gupta, S., Maurya, M. R., Merrill, A. H., Glass, C. K. & Subramaniam, S. Integration of lipidomics and transcriptomics data towards a systems biology model of sphingolipid metabolism. *BMC systems biology* **5**, 26 (2011). URL http://www.ncbi.nlm.nih.gov/pmc/articles/PMC3047436/.

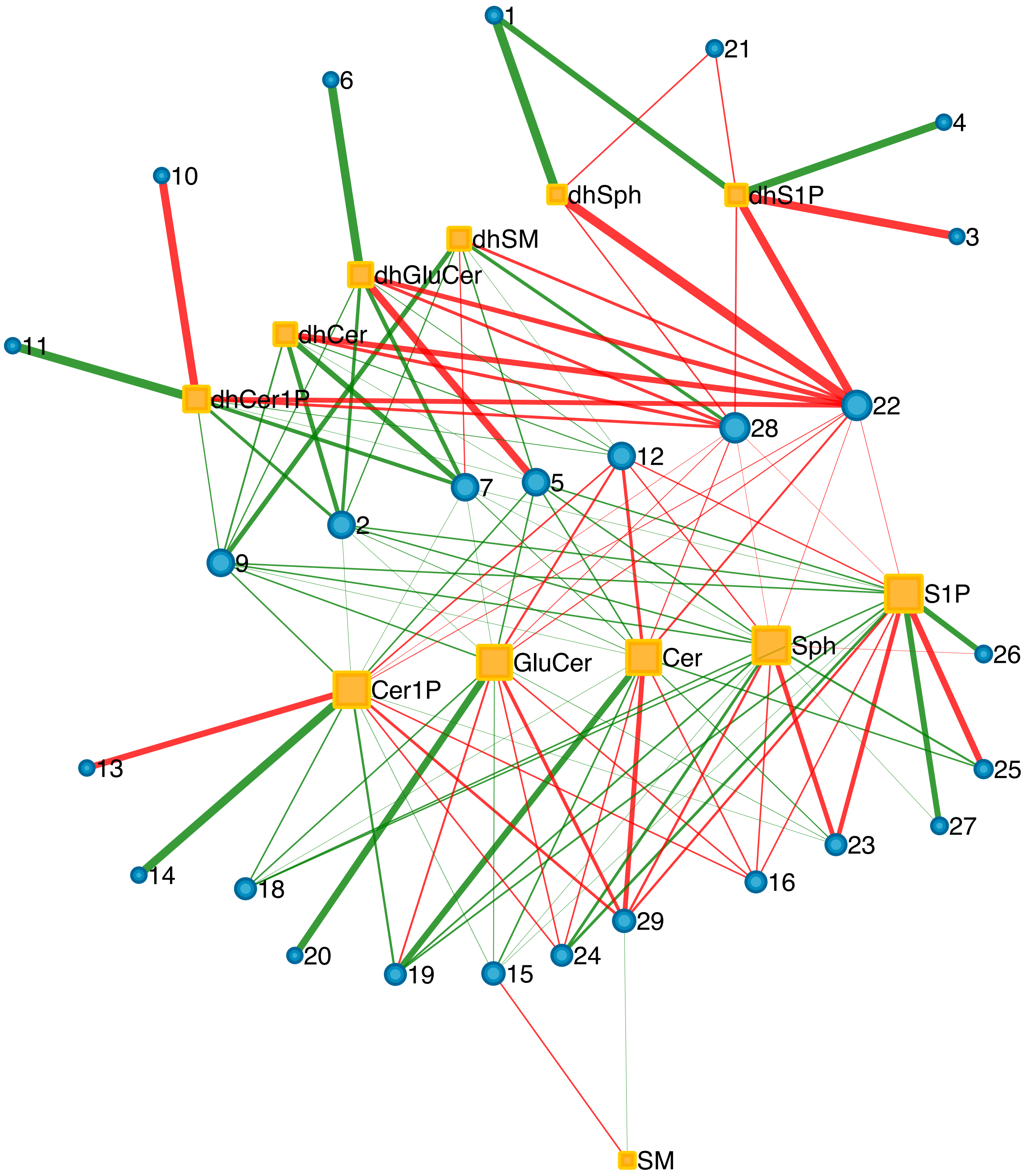

Figure S1. The network of interactions obtained from the sensitivity analysis for rates. Edges are colored in red if at the increase of the rate follows an increase of the concentration of the sphingolipid node, in green if the concentration decreases. The thickness of the edges is proportional to the log_2 of the AUC ratio. In orange rectangles and blue circles, respectively, the sphingolipids and the enzymes are represented. The label of the nodes are the reaction numbers in Tab. S2.

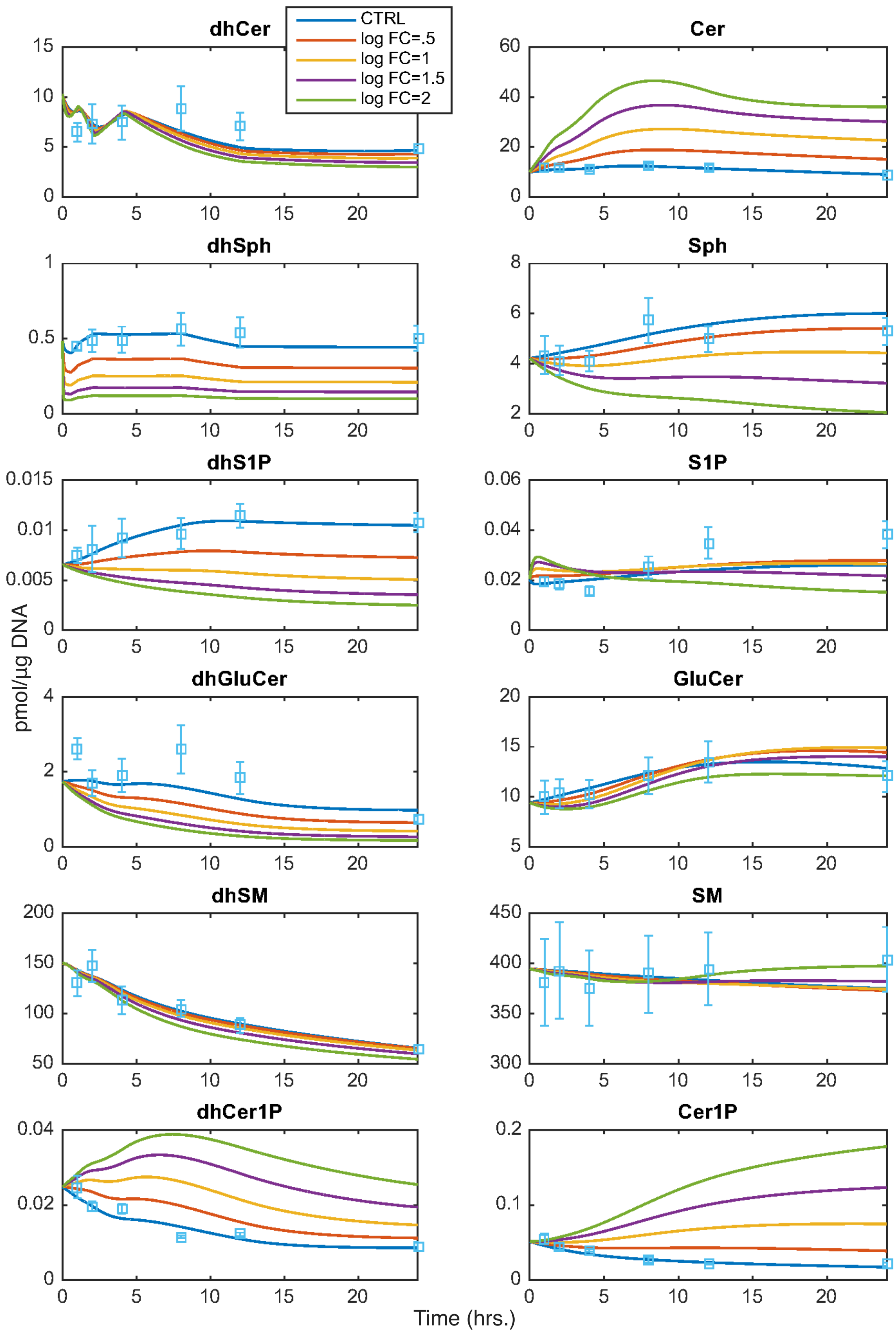

Figure S2. Deterministic simulation of the main metabolites with varying fold changes to observe the effect of the perturbation compared with experimental observations of *ob/ob* mice at 5 weeks. Concentrations measured in pmol/μg DNA. *x-axis*: time in hours.

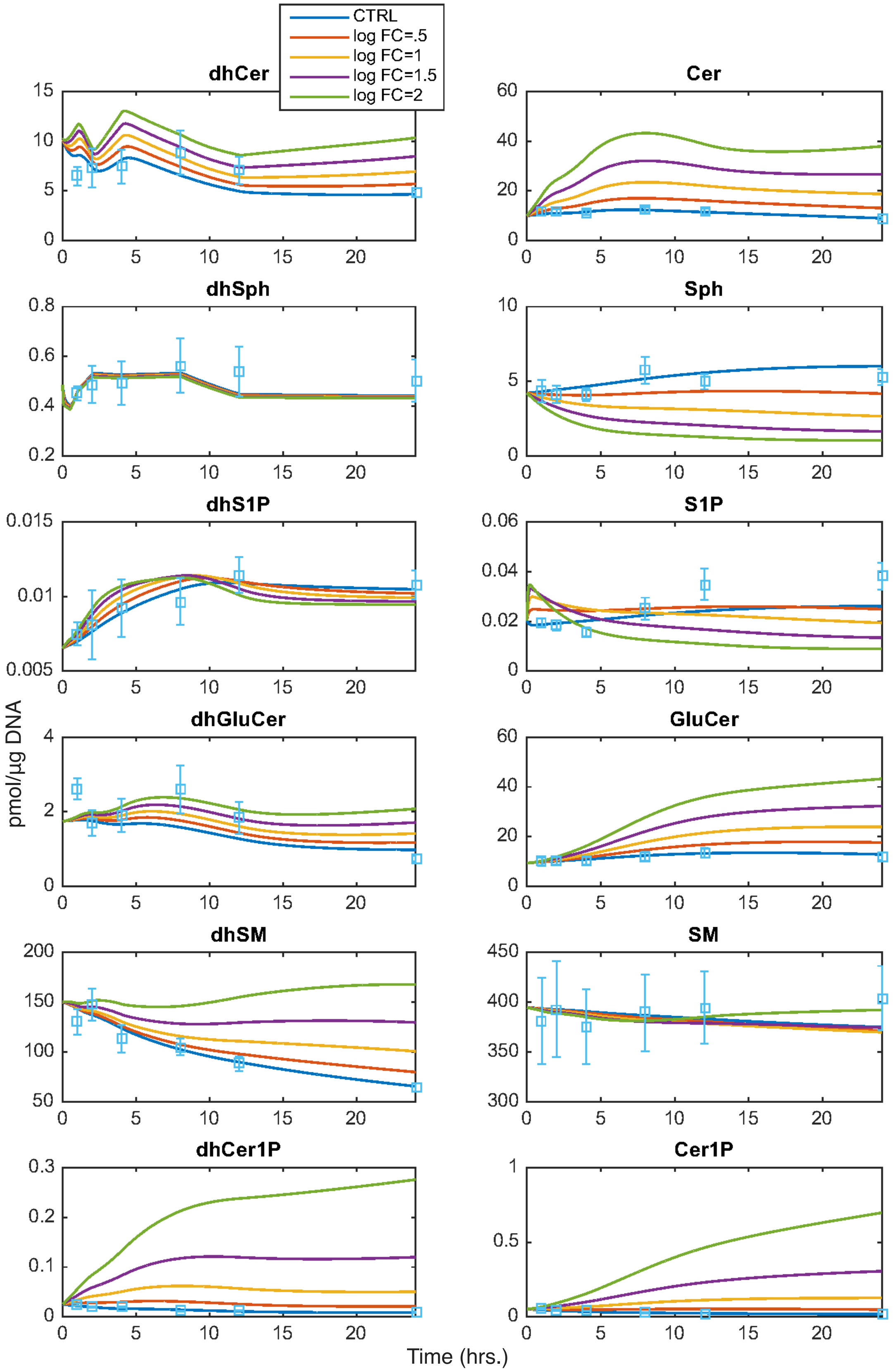

Figure S3. Deterministic simulation of the main metabolites with varying fold changes to observe the effect of the perturbation compared with experimental observations of *ob/ob* mice at 16 weeks. Concentrations measured in pmol/μg DNA. *x-axis*: time in hours.

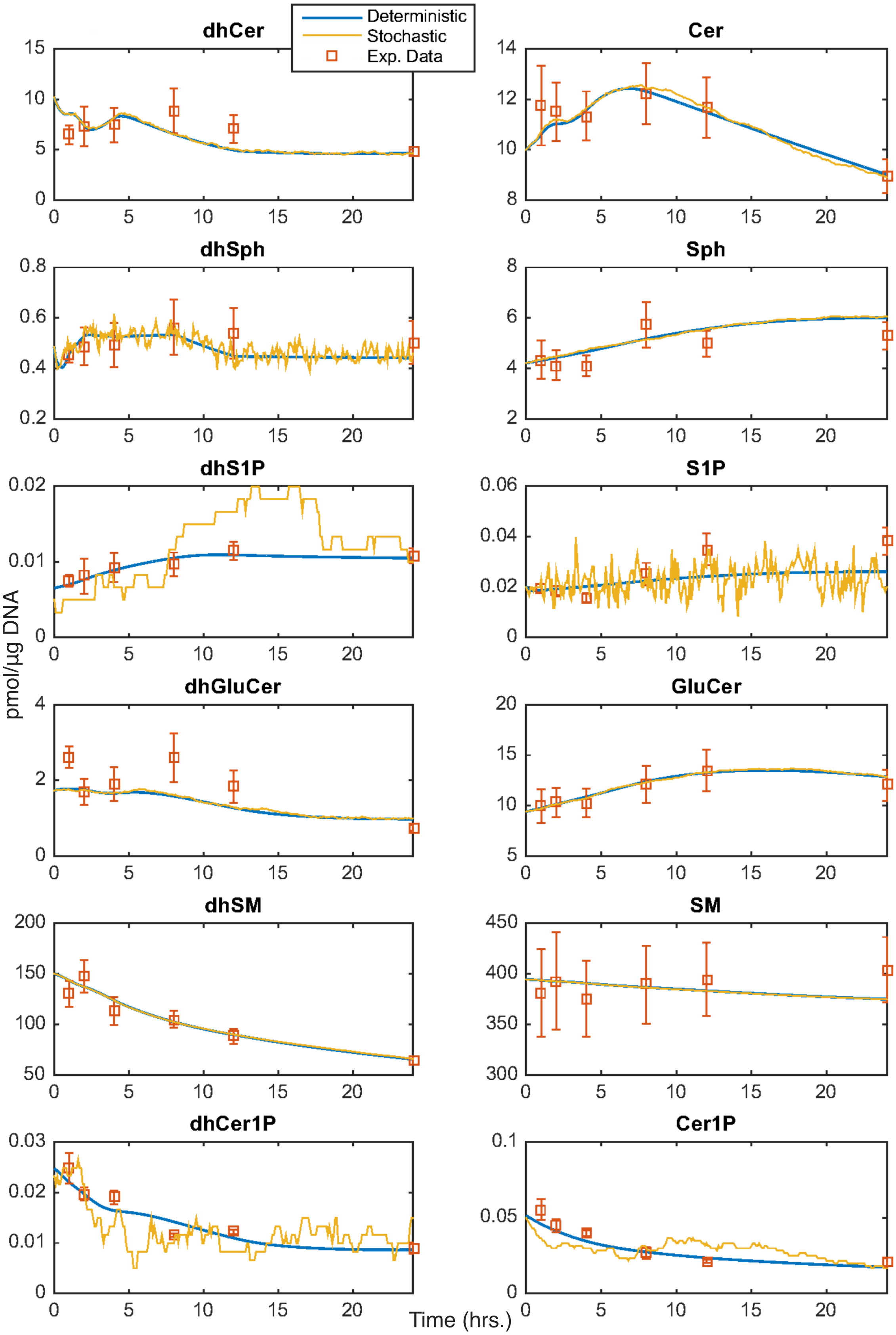

Figure S4. Comparison of the deterministic and stochastic simulation outcomes of the main metabolites with the experimental observation, for RAW 264.7 cells. Note the fluctuation of the stochastic simulations when the abundance of the metabolites is very low, *e.g.* dhCer1P, Cer1P, dhS1P, S1P and dhSph. Concentrations measured in pmol/μg DNA. *x-axis*: time in hours.

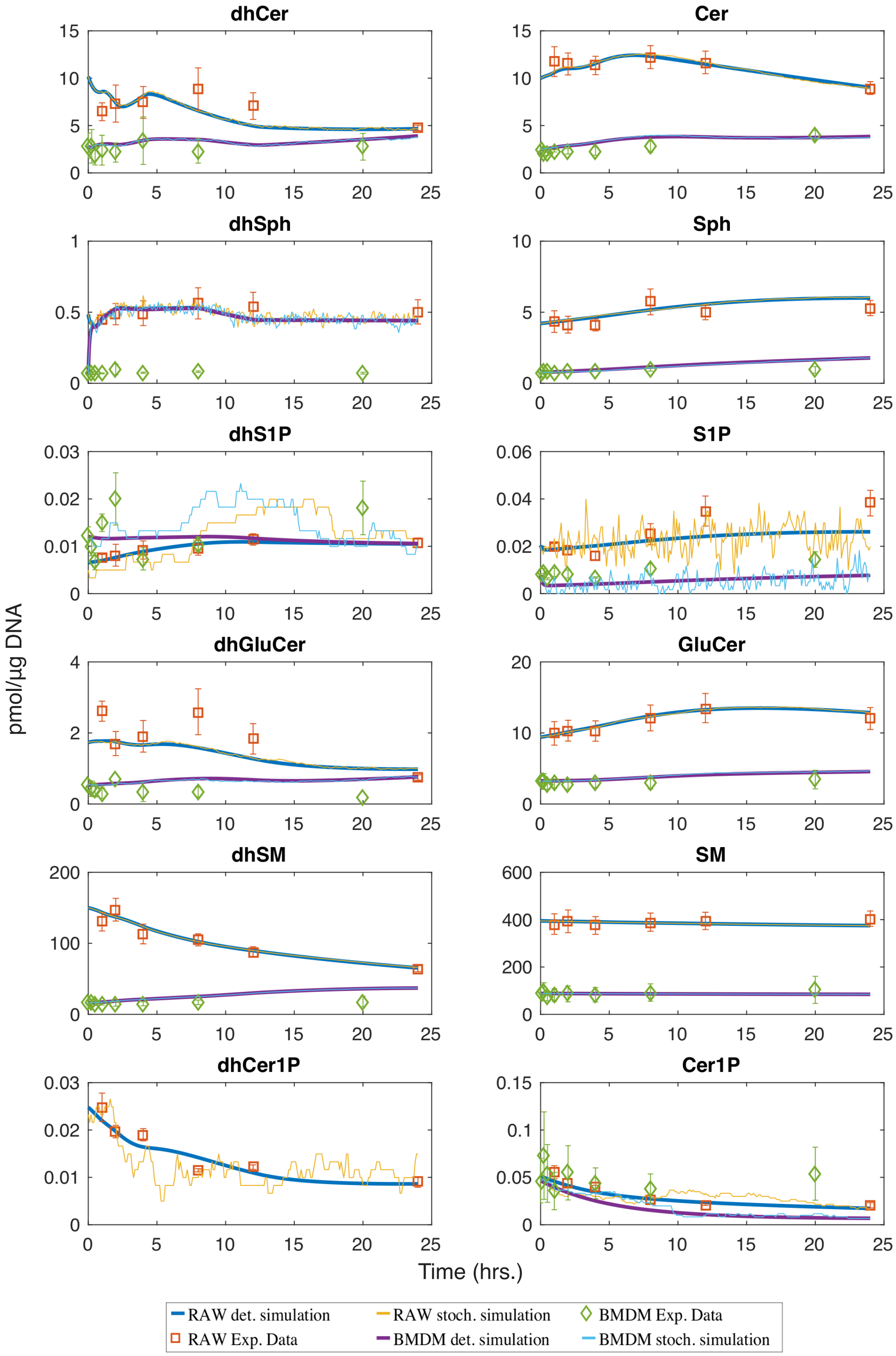

Figure S5. Comparison of the deterministic and stochastic simulation outcomes, for both RAW 264.7 cells and BMDM. The BMDM dataset does not include data for dhCer1P. Concentrations measured in pmol/μg DNA. *x-axis*: time in hours.

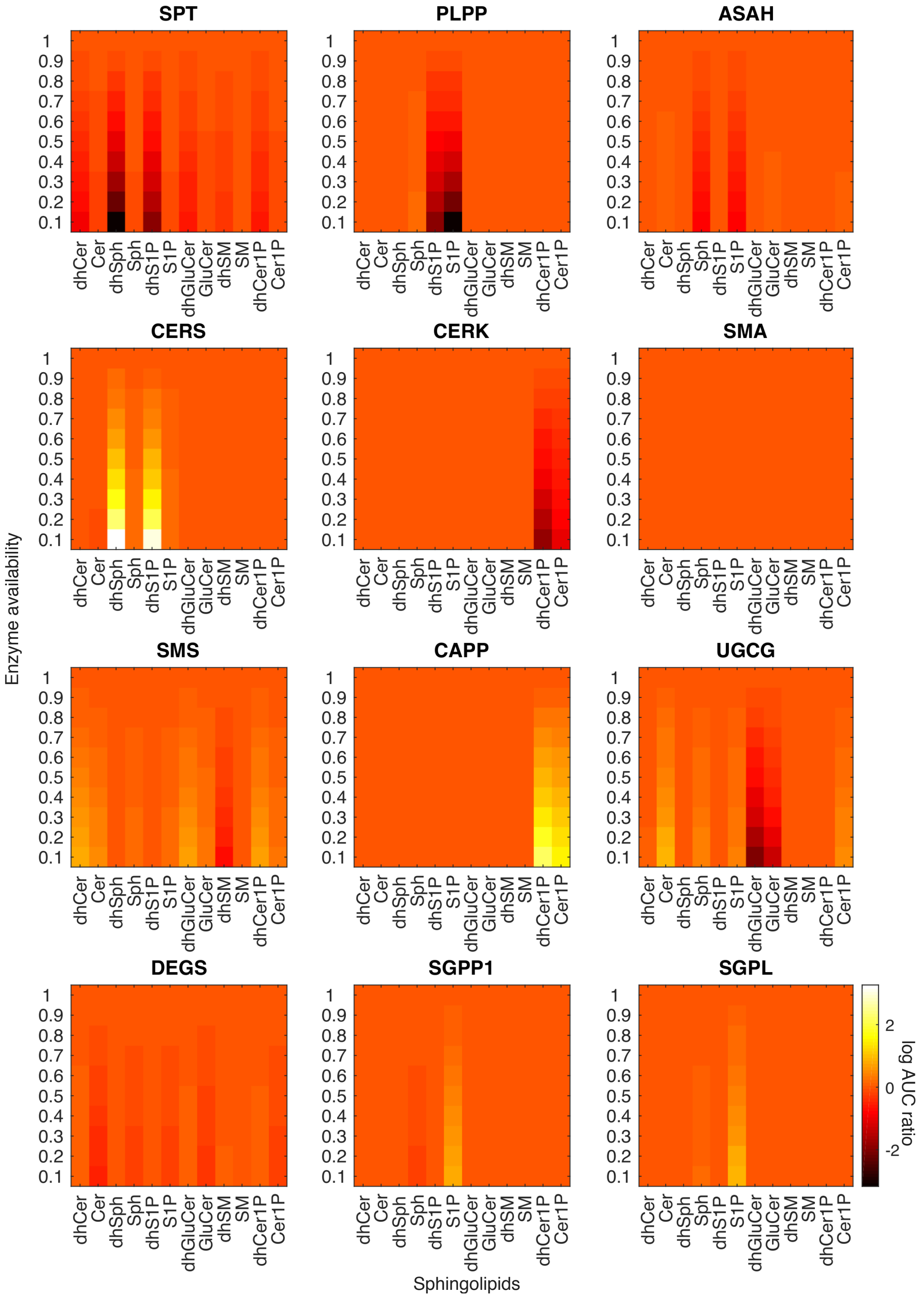

Figure S6. Heatmaps quantifying the effect of the reduced availability of each enzyme on each metabolite. The effect is quantified in terms of $\log_2$ AUC ratio, as defined in the main text.

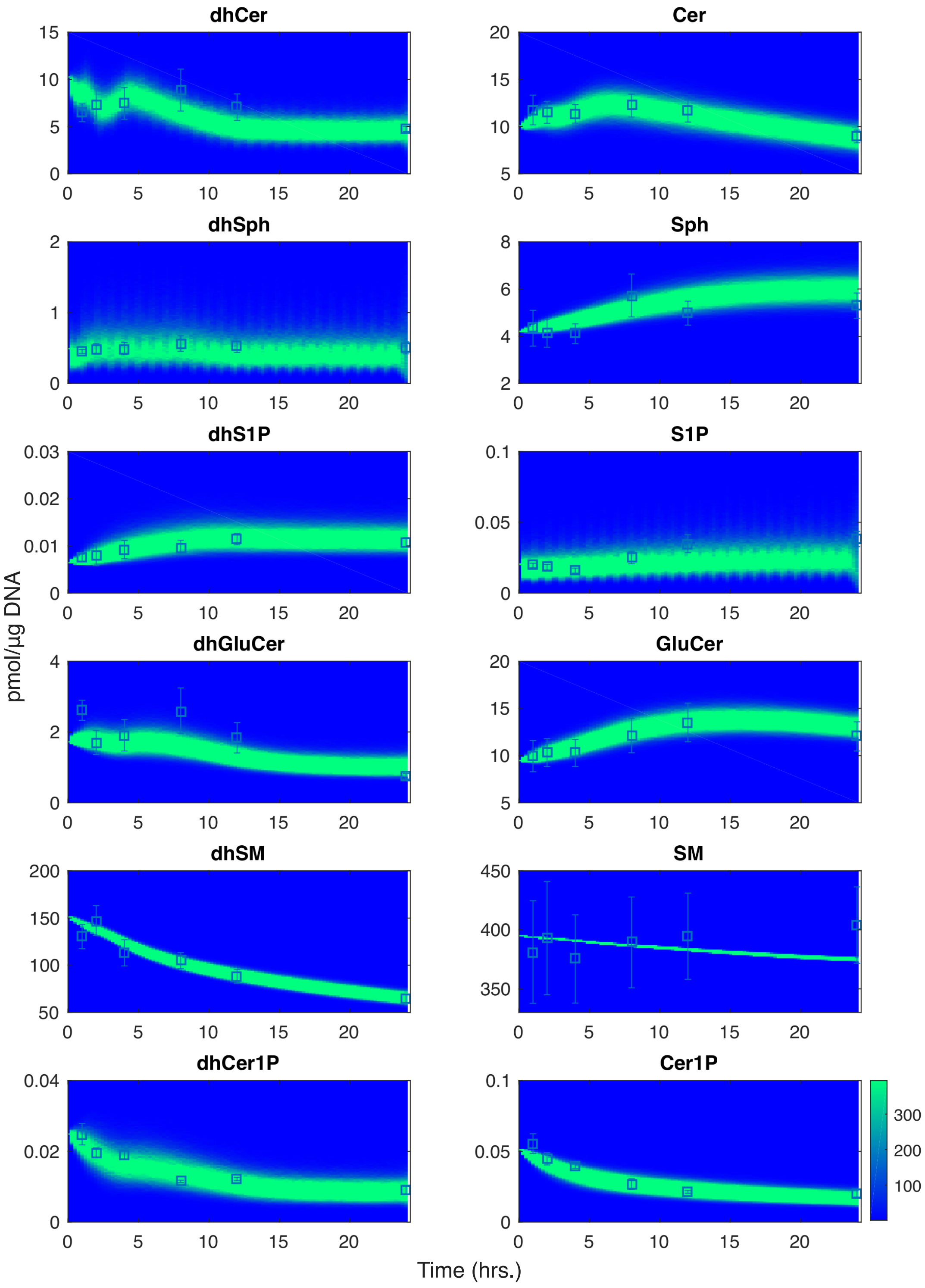

Figure S7. The cumulative output of 10000 simulations under the effect of random perturbations on the fold change of the enzymes, compared with the experimental data. Every hour the enzyme fold changes are randomly varied. The colors are proportional to the number of simulations. Here the greener is the color, the more are the simulations that coincide with that trajectory. The random perturbations are obtained as the sampling of a normal distribution with mean 0 and standard deviation 0.5. Concentrations measured in pmol/μg DNA. *x-axis*: time in hours.

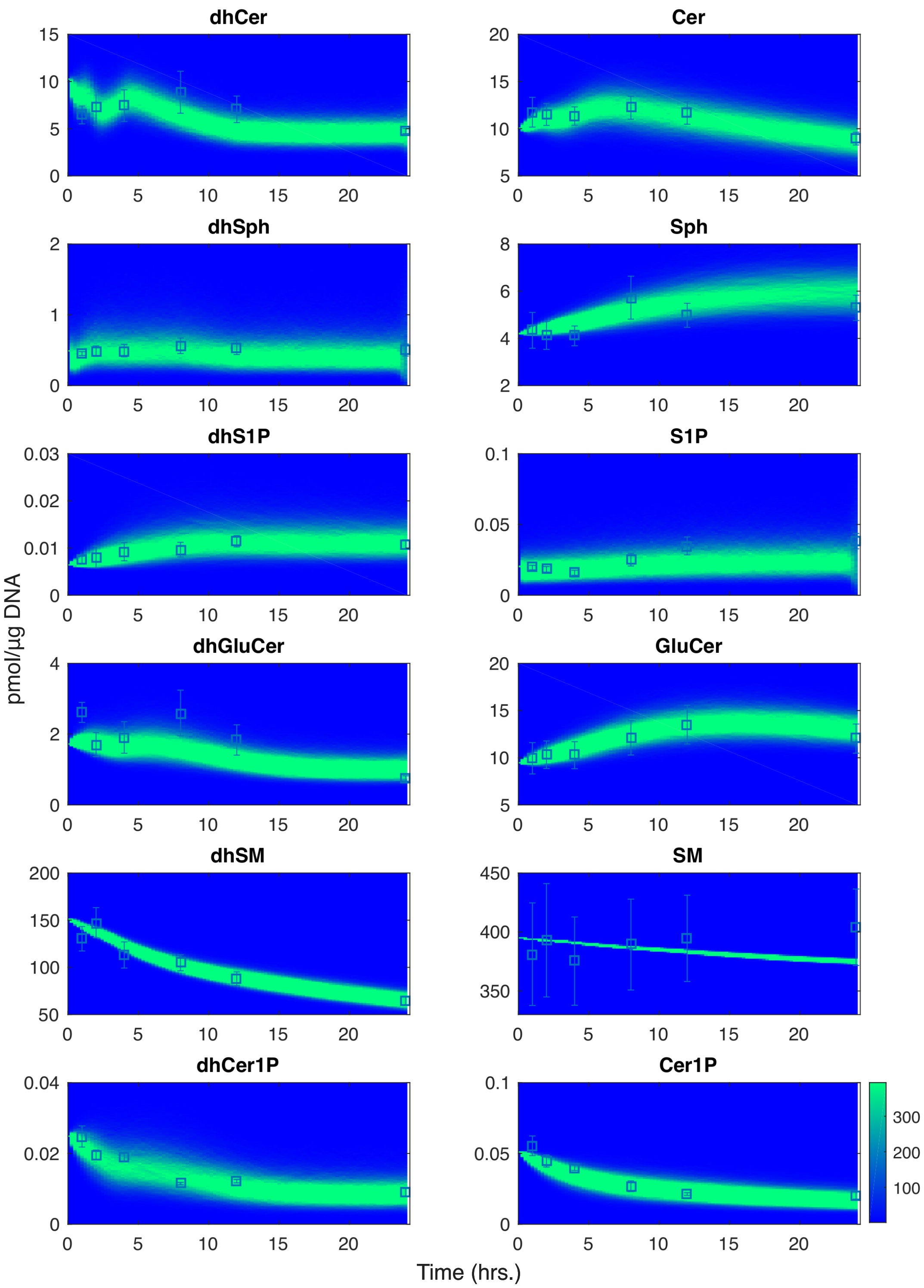

Figure S8. The cumulative output of 10000 simulations under the effect of random perturbations on the fold change of the enzymes, compared with the experimental data. At random time points the enzyme concentrations are randomly varied. The colors are proportional to the number of simulations. Here the greener is the color, the more are the simulations that coincide with that trajectory. The random time points are obtained as the sampling of 15 points from an uniform distribution between 0 and 24. The random perturbations are obtained as the sampling of a normal distribution with mean 0 and standard deviation 0.5. Concentrations measured in pmol/μg DNA. *x-axis*: time in hours.

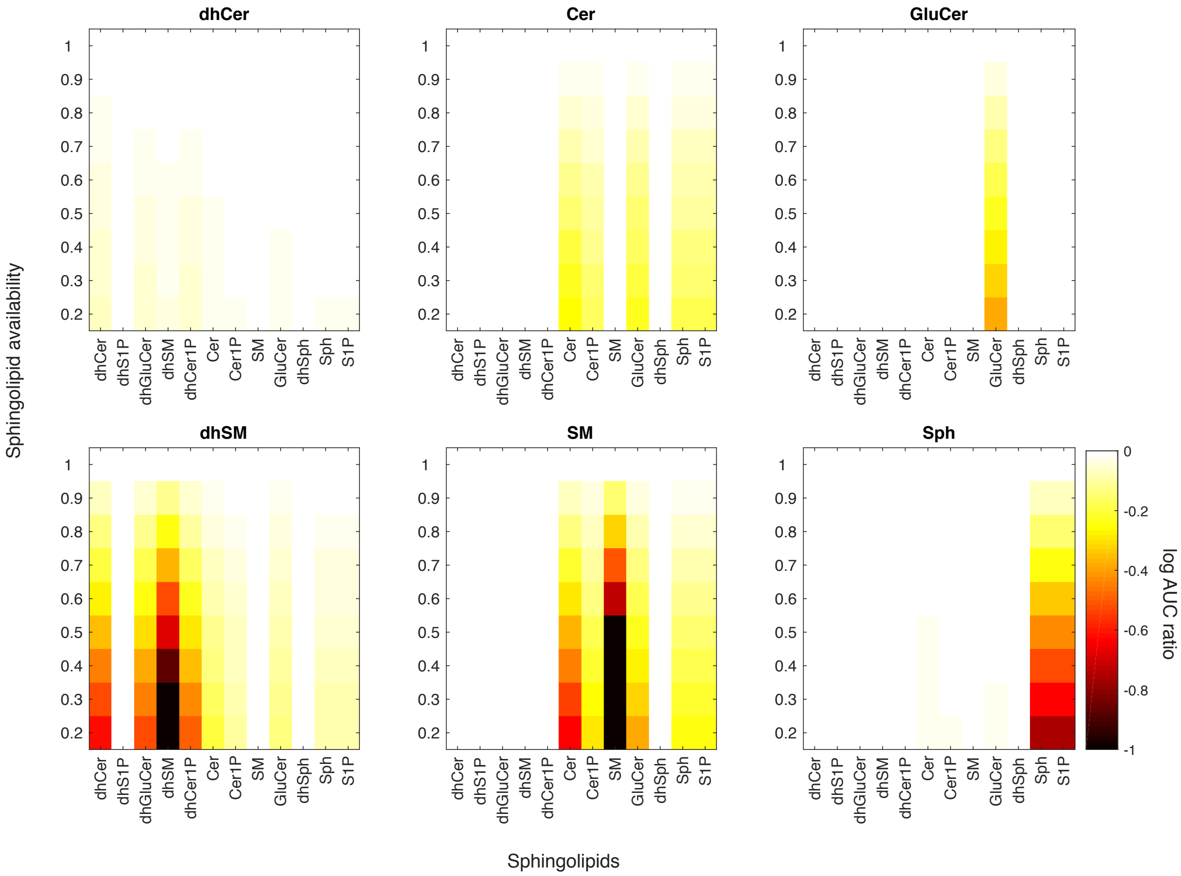

Figure S9. Heatmaps quantifying the effect of the reduced availability of the metabolites due to, for example, impairment in transport between compartments. The reduced effect, displayed in the tiles of each metabolite, is quantified in terms of AUC ratio as defined in the main text.
